# Supplementary material for: Prevalence and determinants of self-medication consumption of antibiotics in children in Iran: A population-based cross-sectional study, 2018–19
Source: PLoS One. 2022 Dec 30;17(12):e0278843. doi: 10.1371/journal.pone.0278843 (PMC9803171; doi:10.1371/journal.pone.0278843)
Supplement: Attachment — Submitted filename: Response to reviewers.docx [file pone.0278843.s002.docx]

Thank you for the comments on our manuscript entitled " Prevalence and determinants of self-medication consumption of antibiotics in children in Iran: a population-based cross-sectional study, 2018-19". We appreciate the suggested modifications and have revised the manuscript accordingly. The revised sections are shown in boldface type and written with different color. The detailed responses to the reviewers’ comments are presented as follows:

**Journal Academic Editor**

Comment 1: Please ensure that your manuscript meets PLOS ONE's style requirements, including those for file naming

**Response: Thanks for your concern upon style requirements, the mentioned naming style requirements of the files will be fulfilled upon re-submission process**.

Comment 2: Please amend your current ethics statement to address the following concerns:
a) Did participants provide their written or verbal informed consent to participate in this study?
b) If consent was verbal, please explain i) why written consent was not obtained, ii) how you documented participant consent, and iii) whether the ethics committees/IRB approved this consent procedure.

**Response: A written informed consent was obtained from the participants of the study.** **The full ethics statement including the ethic codes and criteria of the informed consent will be available in the renewed manuscript in the paragraph entitled “Ethnicity criteria”, located in the first paragraph of the page No 5.**

Comment 3: In your Data Availability statement, you have not specified where the minimal data set underlying the results described in your manuscript can be found. PLOS defines a study's minimal data set as the underlying data used to reach the conclusions drawn in the manuscript and any additional data required to replicate the reported study findings in their entirety. All PLOS journals require that the minimal data set be made fully available.

**Response: In our re-submission, this issued will be addressed via providing a supporting additional file consisting of the minimal data set of the results of our study. Thanks for your concern.**

Comment 4: PLOS requires an ORCID iD for the corresponding author in Editorial Manager on papers submitted after December 6th, 2016. Please ensure that you have an ORCID iD and that it is validated in Editorial Manager.

**Response: The ORCID iD of the corresponding author will be available in the re-submission process and also on the renewed title page.**

Comment 5: Please ensure that you include a title page within your main document. You should list all authors and all affiliations as per our author instructions and clearly indicate the corresponding author.

**Response: Thanks, a renewed title paged including the authors names and affiliations will be uploaded upon the re-submission.**

Comment 6: Please include your full ethics statement in the ‘Methods’ section of your manuscript file. In your statement, please include the full name of the IRB or ethics committee who approved or waived your study, as well as whether or not you obtained informed written or verbal consent. If consent was waived for your study, please include this information in your statement as well.

**Response: The full ethics statement including the ethic codes and criteria of the informed consent will be available in the renewed manuscript in the paragraph entitled “Ethnicity criteria”, located in the first paragraph of the page No 5.**

**Reviewer 1:**

Comments: Positive feedback
The factors considered for the study are effective , valid and as the samples were selected randomly , study was unbiased.
The models and softwares which used were updated and the study considered most of the points which are important in investigating the factors affecting self – medication consumption of antibiotics.
Critical Comments
Others factors such as production of new drugs, the growth of various diseases, access to health information through the Internet could have been considered diring study cause they play important role in health literacy.
Measures to increase the level of awareness and improve community performance about antibiotic use could be suggested.

**Response: Thanks for your elegit and precise comments, the necessary changes are applied to the reviewed manuscript. Proposed corrective measures are mentioned in the “Suggestions” section located as the last paragraph of the page No 10.**

Reviewer 2:

Comments: This study is about self-medication in children, but in the introduction, not enough explanation is given about the target group, for example, explain what are the side effects of self-medication in children? Explain the negative consequences of self-medication in children.
Was the questionnaire valid and reliable? Give a full explanation.
The final conclusion is a very general written and repetitive text of the results.
Add restrictions and suggestions to the article.
At the end of the article, suggest solutions to reduce self-medication in children.

**Response: Thanks for your concern,**

**A more detailed explanation of the side effects of self-medication of antibiotics in children is added to the Introduction section.**

**By referring to the questionnaire, we believe that you are referring to HELIA (Health Literacy for Iranian Adults) questionnaire. Numerous previously published studies suggest that the Health Literacy for Iranian Adults (HELIA) is a reliable and valid instrument for measuring health literacy in Iran.(****1-4****)**

**The conclusion section has been changed, thanks for your apprehension.**

**Suggestions for solutions to reduce self-medication has been added to the renewed manuscript in the paragraph entitled “Suggestions” located**

References

1.	Chahardah-Cherik S, Gheibizadeh M, Jahani S, Cheraghian B. The relationship between health literacy and health promoting behaviors in patients with type 2 diabetes. International journal of community based nursing and midwifery. 2018;6(1):65.

2.	Khoshnudi M, Safari A, Vahedian-Shahroodi M, Sadeghnejhad H, Nejati Parvaz N. The Relationship between Health Literacy and Quality of Life in Nurses of hospitals of Kashmar in 2018. Journal of Health Literacy. 2019;4(1):9-17.

3.	Montazeri A, Tavousi M, Rakhshani F, Azin SA, Jahangiri K, Ebadi M, et al. Health Literacy for Iranian Adults (HELIA): development and psychometric properties. 2014.

4.	Tavousi M, Haeri-Mehrizi A, Rakhshani F, Rafiefar S, Soleymanian A, Sarbandi F, et al. Development and validation of a short and easy-to-use instrument for measuring health literacy: the Health Literacy Instrument for Adults (HELIA). BMC public health. 2020;20(1):1-11.
