# Supplementary material for: Prevalence and determinants of self-medication consumption of antibiotics in children in Iran: A population-based cross-sectional study, 2018–19
Source: PLoS One. 2022 Dec 30;17(12):e0278843. doi: 10.1371/journal.pone.0278843 (PMC9803171; doi:10.1371/journal.pone.0278843)
Supplement: Attachment — Submitted filename: Response to Reviewers.docx [file pone.0278843.s003.docx]

Reviewer #3: Overall, I believe this is an interesting study that can contribute to the literature, but needs further clarification and improvement.
In the introduction,
Sentence starting with “Therefore, by implementing this study..” This sentence must be come before the aim sentence.

Thanks for your concern, the necessary changes were implied.

Sentence starting with “In this study, we asked families …” This information can be transferred to the method section and synthesized with appropriate sentences.

Thanks for your concern, the necessary changes were implied.

In the method, the title of the information about ethics can be corrected as “Ethics” not “Ethnicity Criteria:"

Thanks for your concern, the necessary changes were implied.

When I read this article, I would like to have information about Arak, in a few short sentences.
For example, the socioeconomic status of Arak city, education, industrial city? Are the people ethnically similar? Migration level?

**Arak**  is the capital of [Markazi Province](https://en.wikipedia.org/wiki/Markazi_Province), [Iran](https://en.wikipedia.org/wiki/Iran). At the 2011 census, its population was 526,182, in 160,761 families.[^[3]^](https://en.wikipedia.org/wiki/Arak,_Iran#cite_note-IranCensus2011|00-3)[^[4]^](https://en.wikipedia.org/wiki/Arak,_Iran#cite_note-rc.majlis.ir-4) The city is nicknamed the "Industrial Capital of Iran". As a major industrial city, Arak hosts several industrial factories inside and within a few kilometers outside the city, including the factory of [Machine Sazi Arak](https://en.wikipedia.org/wiki/Machin_Sazi_Arak) and the [Iranian Aluminum Company](https://en.wikipedia.org/wiki/Iranian_Aluminium_Company). These factories produce nearly half of the needs of the country in steel, petrochemical, and locomotive industries. As an industrial city in a developing country, Arak suffers from [air pollution](https://en.wikipedia.org/wiki/Air_pollution).

Moreover, websites containing additional information useful about Arak are enlisted as follows in the references:

(1-4)

It is not clear which tests were applied in the table. a, b Which tests should be shown.

In Table 1, the results of Chi-square and Mann-Whitney U test which were used to compare mean or median values by self-medication of antibiotic consumption are depicted. In Table 2, the results of Univariate and multiple logistic regression models in order to obtain odds ratio (OR) and 95% confidence intervals is shown.

The findings of the study after the regression analysis are actually very important findings. More insight discussion is needed on these.
Interesting findings, for example, in girls and elderly mothers have lower self-medication. Authors should provide explanations to it in their discussion.

Additional explanations are added in the discussion section, thanks.

References

1.	https://en.wikipedia.org/wiki/Arak,_Iran.

2.	https://[www.wikidata.org/wiki/Q212628](http://www.wikidata.org/wiki/Q212628).

3.	https://[www.macrotrends.net/cities/21484/arak/population](http://www.macrotrends.net/cities/21484/arak/population).

4.	https://[www.iranicaonline.org/articles/arak](http://www.iranicaonline.org/articles/arak).
